# Hyperemesis gravidarum and subsequent breast cancer risk

**DOI:** 10.1038/sj.bjc.6600605

**Published:** 2002-10-21

**Authors:** G Erlandsson, M Lambe, S Cnattingius, A Ekbom

**Affiliations:** Department of Medical Epidemiology, Karolinska Institutet, PO Box 281, SE-171 77 Stockholm, Sweden; Clinical Epidemiology Unit, Department of Medicine, Karolinska Hospital, SE-171 76 Stockholm, Sweden; Department of Epidemiology, Harvard School of Public Health, Boston, Massachusetts, MA-02115, USA

**Keywords:** breast neoplasms, hyperemesis gravidarum, case–control studies

## Abstract

Both parity and a young age at first pregnancy are associated with a reduction in breast cancer risk. The hormones involved in this process are not fully investigated. Human chorionic gonadotropin is a placental hormone, which in rats and in human breast cells *in vitro* has been shown to prevent against breast cancer. Hyperemesis, a severe nausea combined with vomiting during pregnancy, is associated with increased levels of human chorionic gonadotropin. We investigated the possible relationship between hyperemesis and subsequent breast cancer risk in a case–control study based on registry data. Among 13 079 breast cancer cases and 34 348 individually matched controls we found 148 cases and 405 controls who had been hospitalised for hyperemesis. Hyperemesis was not associated with breast cancer risk (adjusted odds ratio 1.05, 95% confidence interval 0.86–1.27), and similar risks were observed regardless of age at diagnosis, number of hospitalisations for hyperemesis or time of follow-up. Our results do not support the hypothesis that human chorionic gonadotropin is responsible for the protective effect of pregnancies upon breast cancer risk.

*British Journal of Cancer* (2002) **87**, 974–976. doi:10.1038/sj.bjc.6600605 www.bjcancer.com

© 2002 Cancer Research UK

## 

The biological mechanisms by which childbearing reduces breast cancer risk are not fully understood. Human chorionic gonadotrophin (hCG) is a pregnancy specific hormone, produced by trophoblasts. In animal models, hCG has been shown both to prevent and to inhibit growth of breast cancer induced by 7,12-dimethylbenz-*a*-anthrazene (DMBA) (Russo and Russo, 2000). Likewise, hCG has been shown to inhibit growth of human breast cancer cells *in vitro* (Srivastava *et al*, 1998). Based on these findings, hCG has been proposed as a potential chemopreventive agent against breast cancer, especially if administered to young, nulliparous women (Rao, 2000).

Observational data on exposure to hCG and subsequent breast cancer risk are sparse. One study found a significant decrease in breast cancer risk among women who had been administrated hCG as part of an infertility treatment or as a weight-loss regimen (Bernstein *et al*, 1995). On the other hand, two studies found small increases in risk among women with a previous diagnosis of hydatidiform moles, a benign placental proliferative disease linked to elevated levels of hCG (Olsen *et al*, 1999; Erlandsson *et al*, 2000).

Hyperemesis gravidarum, namely nausea combined with persistent vomiting during pregnancy, is linked to increased levels of hCG compared to normal pregnancies the levels being proportional to the degree of nausea (Goodwin *et al*, 1992). Thus, women with a history of hyperemesis form a suitable group in which to study effects of exposure to high hCG levels.

We explored the possibility of an association between inpatient care for hyperemesis gravidarum and the subsequent risk for breast cancer using Swedish registry data, which also allowed us to adjust for confounders, such as parity and age at first birth.

## MATERIALS AND METHODS

### Data sources

Data for the present study was obtained from four population-based registries: the Swedish Cancer Register, the Swedish Register of Births, the Swedish In-patient Register, and the Swedish Medical Birth Register. Information from these registries can be merged using the unique 10-digit identification number assigned to all Swedish residents at time of birth or first residency.

In the Swedish Cancer Register, information on all incident cancers in Sweden has been recorded since 1958. Notification of cancer to the Swedish Cancer Register is mandatory both for clinicians and pathologists, resulting in an estimated completeness of over 98% (Mattsson *et al*, 1985).

The Swedish Register of Births is part of the Swedish population statistics and has recorded information on all births since 1961, including sex of the child, multiple births, and area of residence of the mother.

The Swedish In-Patient Register contains diagnoses and dates of admission of all hospitalised patients in Sweden. The registry was established in parts of Sweden in 1964, and the coverage has since been gradually expanded to include all Sweden from 1987 onwards, estimated now to be 99% complete (Swedish National Board of Health and Welfare, 1999).

The Swedish Medical Birth Register has recorded information on all births since 1973, as well as background information on the pregnancy and the mother. The registry is notified by the delivery units and the completeness of the register has been estimated to be close to 100% (Cnattingius *et al*, 1990).

### Study population

The study population consisted of all Swedish women who had given birth between 1st January, 1964 and 31st December, 1999 while living in a region covered by the Swedish In-patient Register.

Potential cases were identified through a linkage between the Swedish Cancer Registry and the Swedish Registry of Births and included all women who had given birth between 1st January, 1964 and 31st December, 1999, and had a subsequent diagnosis of primary breast cancer through 31st December, 1999. This resulted in data on 30 419 women.

Four controls per case were initially selected and individually matched to cases on year of birth. Controls were randomly selected from the Swedish Registry of Births and included women who had given birth in Sweden during the study period. Controls were eligible if they were alive, had not emigrated, and did not have a diagnosis of breast cancer by the date of diagnosis of the case.

Data on diagnoses of hyperemesis as well as a number of confounding variables were matched to cases and controls from the Swedish In-patient Register and the Swedish Medical Birth Register for all pregnancies covered by the two registries. To increase precision, we excluded cases and controls who had not been resident in a region covered by the Swedish In-patient Register during any pregnancy. Due to the matched design also the corresponding controls to excluded cases were excluded. This left us with data on 13 079 cases and 34 348 matched controls, a total of 47 429 women.

### Statistical methods

Relative risks were estimated by calculation of odds ratios (OR) with 95% confidence intervals (95% CI) using conditional logistic regression for matched case–control studies.

To evaluate differences in effect on pre- and post-menopausal breast cancer, we made sub-set analyses of cases with an age at diagnosis of above and below 50 years. To reveal potential long-term effects of hyperemesis we also made subset analyses by time since hyperemesis.

Height was evaluated as a potential confounder in a subset analysis consisting of all risk-sets where height was available for the case and at least one control (2878 cases with a total of 7137 controls).

## RESULTS

Mean age at diagnosis of breast cancer was 45 years. Of the cases, 67% were below 50 years of age at diagnosis and were in the analysis considered as pre-menopausal. Compared with controls, cases had on average fewer births, a higher age at first birth, and were taller. There were no differences in offspring gender or proportion of multiple births among cases and controls. The characteristics of cases and controls are shown in Table 1

**Table 1:**
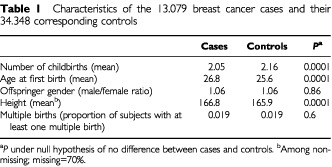
Characteristics of the 13.079 breast cancer cases and their 34.348 corresponding controls

.

A diagnosis of hyperemesis during at least one pregnancy was reported for 148 cases and 405 controls, resulting in an odds ratio of 1.05 (95% CI 0.86–1.27) after adjustment for parity, age at first birth and the number of pregnancies within a region covered by the in-patient registry (Table 2

**Table 2:**
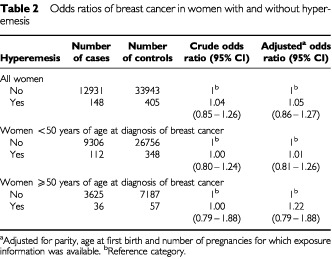
Odds ratios of breast cancer in women with and without hyperemesis

). Further adjustments for offspring gender and multiple births did not alter the estimates. Height was in the subset analysis not found to influence the risk estimate (data available on request). A history of hyperemesis was not associated with breast cancer below 50 years of age (adjusted OR=1.01), but at 50 years of age or later this was slightly higher, OR=1.22, although not significantly increased (95% CI 0.79–1.88).

There was no trend in breast cancer risk with number of pregnancies with hyperemesis. Equally, no trend in risk was observed depending on time between hyperemesis and diagnosis of breast cancer (Table 3

**Table 3:**
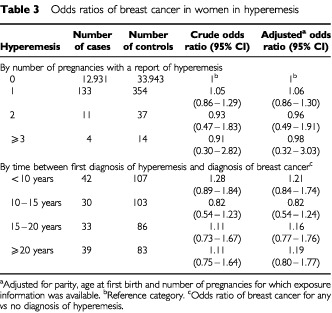
Odds ratios of breast cancer in women in hyperemesis

).

## DISCUSSION

We found no altered risk of breast cancer following one or more diagnoses of hyperemesis. The lack of association was independent of length of follow-up. We found no decrease in risk, either for premenopausal or for postmenopausal breast cancer. A slight but not significantly increased risk was observed for postmenopausal breast cancer.

At least two previous studies have examined possible associations between nausea during pregnancy and subsequent risk of developing breast cancer. One (Troisi *et al*, 1998) found a decreased risk following one or more pregnancies with self-reported nausea, whereas the other (Enger *et al*, 1997) instead observed a slight risk increase after hospital care for hyperemesis. The reasons for the conflicting results are unclear. Both studies were based on retrospective interview data, a design that is sensitive to differential reporting between cases and controls. In our study, differential misclassification is not an issue, since information on hyperemesis was collected from an independent source prior to onset of disease.

Only a little more than 1% of subjects had received inpatient care for hyperemesis. However, the study was large enough to provide reasonably large numbers of cases and controls exposed to hyperemesis. Levels of hCG increase with severity of nausea (Goodwin *et al*, 1992), and hyperemesis requiring hospital care is probably associated with severe nausea. Thus, our choice of hyperemesis as proxy variable for hCG exposure increased the difference in exposure to hCG between exposed and non-exposed women. In contrast, also including milder forms of nausea as exposures of interest would have diluted the exposed population with women suffering from nausea by non-hormonal reasons.

Parity and young age at first birth are strong protective factors against breast cancer (Kelsey *et al*, 1993). A number of possible mechanisms have been proposed to explain this protection. Childbearing, especially a first pregnancy, has been postulated to cause a permanent modification of the biological characteristics of the breast. Following a pregnancy, breast epithelial cells differentiate, a process that makes them more resistant to neoplastic transformation (Russo *et al*, 1982). The risk of malignant change may also be reduced through a slowing down in the subsequent rate of cell proliferation, and possibly also by a decrease in the carcinogen-binding capacity (Russo and Russo, 1994). It remains unclear, however, if these mechanisms can fully account for the lowered breast cancer risk imparted by a pregnancy. Furthermore, the hormones responsible for the postulated mechanisms are not yet identified (Kelsey *et al*, 1993).

Our results suggest that pregnancies do not protect against breast cancer through hCG, at least not in a dose-dependent manner. As women with hyperemesis are exposed to high levels of hCG, they provide one of the few human populations in which to study effects of this hormone. Apart from increases in serum hCG, hyperemesis has also been linked to raised levels of circulating oestrogens, particularly oestradiol (Eliakim *et al*, 2000). Given that oestradiol would increase breast cancer risk, this could theoretically conceal a true protective effect of hCG. Oestrogens in pregnancy-like doses have, however, been shown to protect against breast cancer in rats in a manner similar to hCG (Rajkumar *et al*, 2001). Thus, opposing effects on breast cancer risk by the different hormones seem unlikely. Hyperemesis has also been linked to reproductive history, twinning and to an altered sex ratio of the offspring (Askling *et al*, 1999; Eliakim *et al*, 2000), all of which could possibly affect breast cancer risk (Kelsey *et al*, 1993; Hsieh *et al*, 1999). All these factors were accounted for in our study, and adjustments had no marked impact on the estimates.

Only the long-term breast cancer risk is reduced by pregnancies (Lambe *et al*, 1994). A transient increase in breast cancer risk after hyperemesis would thus not rule out the possibility of a long-term protection. However, we did not observe any decrease in risk even in the group with more than 20 years of follow-up.

In conclusion, we found no association between hyperemesis and subsequent breast cancer risk, and therefore no support for a protective effect of hCG. In spite of the promising results from animal and *in vitro* studies, confirmation is required before hCG can be tested as a preventive agent against breast cancer.
